# Enhancing academic integrity in a UAE safety, security defence emergency management academy – the Covid- 19 response and beyond

**DOI:** 10.1007/s40979-022-00110-3

**Published:** 2022-08-04

**Authors:** Amanda Davies, Rami Al sharefeen

**Affiliations:** Faculty of Resilience, Rabdan Academy, Abu Dhabi, UAE

**Keywords:** Police officer education, Assessment authenticity interviews, Online-based (internet) assessment, Academic integrity policy

## Abstract

Globally, academic integrity and misconduct is a continuing conundrum for education institutions. Whilst the online (internet based and remote) delivery of education is not new, the onset of Covid-19 with accompanying health and safety limitations and the consequential rapid transition to emergency online delivery of education has, for many, exacerbated the need to focus on emerging potential for new forms of student academic misconduct i.e., e-dishonesty. This paper presents the strategies developed by a higher education institution specializing in university courses for safety and security professionals in the United Arab Emirates to address academic integrity during Covid-19 and beyond. The fundamental approach draws on lessons learnt from across the education community and focuses on engaging a comprehensive whole of Academy (faculty and student) commitment to a high standard of academic integrity. The research investigated the outcomes of the academic integrity interventions in a total student population of 631. The early trending results of the strategies are encouraging, whilst monitoring of the ever-changing academic assessment landscape is pivotal to ensure these early trends are validated and sustained. The strategies developed and deployed by the Academy are replicable and offer a contribution to the demanding and complex challenge of establishing academic integrity within educational institutions across the world.

## Introduction

To enable contextualization of the later discussion, it is helpful to offer a brief description of Rabdan Academy. The Academy is a government-owned, education institution established to coordinate and enhance learning outcomes for organisations and individuals in the safety, security, defence, emergency preparedness and crisis management sectors in the Emirate of Abu Dhabi. The Academy provides learning in a dual approach, combining academic and vocational education in one place whilst recognizing prior learning and experience and providing transferable credit from course to course and job to job. Primarily students are drawn from the military and police, either commencing their Diploma, Bachelor or Masters studies with operational experience or transitioning from completion of their high school studies. The Academy offers (a) Diplomas in Policing and Security and Crime Scene; (b) Bachelor degrees in Policing and Security, Homeland Security, Integrated Emergency Management, Business Continuity Management, and (c) Masters Degrees in Intelligence Analysis and Policing and Security Leadership. All graduates of Rabdan Academy return to their respective police or military organisations to continue their careers. Of note, Arabic is the native language for the students, their studies at Rabdan Academy are conducted in the English language (with the exception of the Crime Scene Diploma which is delivered in the Arabic language). An important factor is 99% of students are on scholarships at the Academy from their respective stakeholder Military and Police entities. This factor influences the student’s motivation to not only pass their course, also to achieve high grades. Failure has substantial financial, status and morale impacts on the student. The education program for police students is referred to as police education as the graduates become police officers as members of the Abu Dhabi Police, their program of study is a Bachelor of Policing and Security or Diploma of Policing and Security or Masters of Policing and Security Leadership.

Prior to the onset of the Covid-19 pandemic in the United Arab Emirates (UAE) in the Spring of 2020, the Academy had not previously delivered courses as a distance / remote education approach utilizing the internet (referred to here as ‘online’) environment. The transition to all courses to be delivered via the internet, either live streaming in real time or pre-recorded lectures and instructional material, was rapid and involved developing both faculty and students in this new mode of learning delivery. In association turning attention to managing academic integrity in this new learning environment was urgent.

As discussed in the following, the Academy approached the design and development of Academic Integrity policies and procedures for this unfamiliar learning environment based on engaging both faculty and students. This paper presents the design, management, and results of this ‘whole of Academy’ approach coupled with the specific academic integrity mitigation strategies. In particular, the underpinning concept of proactive preparation of both students and faculty with knowledge of the working application of the policies and protocols, not only for online examinations, but also for submission via the online (internet) of written essays and assessments. The process across five terms enabled lessons to be learnt and adjustments to processes and protocols for continuous quality enhancement. An important aspect of the Academy approach was to recognise and support the student potential learning curve through a scaffolded process of assessment submission and plagiarism monitoring and a range of academic integrity violations based on severity and recidivism.

## Literature review

The literature suggests education institutions across the globe continue to be challenged in establishing effective academic integrity policies and processes within their respective institutions (Arora 2020; Clark et al. 2020; Parnther 2020; Sefcik, et al., 2019). Whilst this conundrum is not new as discussed by Sedir and Maxwel 2020; Noorbehbahani et al. 2022; Pell 2018, the rise of emergency online education delivery due to the global Covid-19 pandemic has increased the focus and urgency of developing reliable and sustainable measures (Reedy et al. 2021a, 2021b). The size of the dilemma is appreciatively, immeasurable; it is evident in the literature the rapid transition to online learning and assessment in the midst of the global Covid-19 pandemic has created concern as to the influence on the increased rate of academic integrity violations (Monico 2021; Goff et al. 2020; Reich and OMN 2020). The work of Eaton (2020) in discussing the challenges for maintaining academic integrity during the emergency transition to online learning in response to Covid-19, signposts concern in two key fields (a) from faculty who ‘resist adapting assessment practices’ and (b) the ever-present contract cheating industry poised to take advantage of vulnerable students ‘caught in the middle of a higher education system that has not fully adapted to online learning’ (p.83). The work of Nguyen et al. 2020 similarly focused on the role of adapting online assessment design to mitigate potential for academic misconduct during Covid-19. In the context of this paper, it is the plethora of literature that has emerged discussing, debating, and proffering strategies to address the challenge of (a) developing strategies to support student and faculty understanding of expectations, violations, and penalties for academic misconduct (see Mathrani et al. 2021; Gamage et al. 2020) and (b) building whole of institution resilience (see Reedy et al. 2021a, 2021b; Khan et al. 2020). The work of Sefcik et al., 2019 and Gamage et al. 2020 signposted the increasing global recognition of the importance of academic integrity and the emerging strategies being supported beyond institutional levels to national government levels. The 2019–2021 period has witnessed examples of the extent to which governments have supported initiatives to address the situation as illustrated by the Australian Parliament sanctioning the Tertiary Education Quality and Standards Agency Amendment (Prohibiting Academic Cheating Services) Bill 2019 (Australian Parliamentary Handsard, 2019 aph.gov.au). The intent of the Bill to deter academic cheating services by the following:

- make providing and advertising academic cheating services subject to offences and civil penalty provisions under the *TEQSA Act* and
- preventing and minimizing the use and promotion of academic cheating services to the responsibilities of Australia’s higher education regulator, the Tertiary Education Quality and Standards Agency (TEQSA).

Similarly, media outlets are reporting draft laws under consideration in China to address academic misconduct (Xinhuanet 2021). The report suggests China is considering a law to regulate academic awards to guard against plagiarism, cheating and other misconduct, with penalties extending to revoking degrees if students are found to have committed academic misconduct, including plagiarism, forgery, and data fraud in their dissertations or practical achievements. In 2021, the Quality Assurance Agency for Higher Education in the United Kingdom reported government ministers pledging support for higher education providers to combat the threat of essay mills and academic misconduct through application of the Academic Integrity Charter. Matthews (2019) writing for the Times Higher Education reports the Balkans leading a drive to criminalise academic misconduct as Montenegro becomes one of the first countries in the region to outlaw not only plagiarism, but also donation of authorship and fabrication of research results (p.1).

In concert with academic institutions around the world, academic institutions in the United Arab Emirates are similarly challenged in safeguarding academic integrity. A systematic review undertaken by Khan et al., in 2019 of the universities’ open-source academic integrity policies in the UAE provides insight into the diverse approaches to this academic issue across the UAE higher education. At the time of the systematic review, (Khan et al. 2019 p. 2) reported:

In accordance with the UAE Vision 2021, the country has become an attractive education hub for students from around the world in recent years with over 25 international branch campuses from USA, UK, Australia, Canada, India, Iran, to name a few, and hundreds of other institutions housing hundreds of students from over 80 nationalities. As a young sector, the education sector in the UAE is yet to have established sector standards and best practices that can be found across campuses in the UAE for academic integrity. Some universities have academic integrity policies, some don’t.

Khan et al. 2019, p. 6 further suggest a more comprehensive review of UAE higher education institutional approaches to policies and procedures regarding academic integrity would offer opportunity to develop exemplar good practices. This paper offers insight into the adoption and outcomes of a holistic approach to building academic integrity and mitigating academic integrity transgressions in a United Arab Emirates higher education institution. The actions associated with academic dishonesty are discussed widely in the literature and the work of Bretag et al. (2019a, 2019b) which is a helpful example offering a valuable summary of the range of academic integrity transgressions. Bretag et al. (2019a, 2019b) suggests transgressions include a range of sharing papers, to ghost writing and or buying of assessments and all variations on this spectrum, underpinned by the increasing proliferation of individuals and organisations advertising writing services and advice for avoiding integrity security gateways. It is this interpretation and understanding of the range of acts of academic integrity that have been included within the development of mitigating strategies at Rabdan Academy. In parallel, designing academic integrity procedures to meet the transition to online (internet) based examinations for students studying off campus due to Covid-19 was a new domain to be governed. Harris et al. (2020) refer to the Covid-19 pandemic as creating online (internet based) exams as a critical consideration for higher education and draws with it the concern of faculty and students with academic integrity of online assessment – e-dishonesty. The term e-dishonesty was coined in the work of (Sendag et al. 2012, p.1) in reference to academic dishonesty associated with online (internet based) violation of academic integrity. As the Academy developed policies and procedures to meet these challenges, the works of Liang and Maddison 2021; Reedy et al. 2021a, 2021b; Thacker et al. 2020; Chankova 2020; resonated with the notion of a holistic whole of education community approach to guide the design of supportive strategies. Further influence on the development of an approach to academic integrity is found in the work of Sefcik et al. 2019 which revealed:

…that, in many cases, current academic integrity education programs appear to lack comprehensive information on values, the potential risks to integrity and the pitfalls of assessment outsourcing. Instead, curricula tended to focus on plagiarism, student responsibilities and referencing (p.1).

In addition, the work of Freeman et al. (2014) advocating for students to be at the center of the learning process to encourage engagement with academic integrity have informed on the approach undertaken by Rabdan Academy in this field. Interestingly, as the evaluation of Rabdan Academy’s approach was in progress, the work of Dyer et al. (2020) emerged suggesting the academic environment may influence student response to academic integrity, such a suggestion resonated with the Rabdan Academy approach. In a similar theme, Turner et al. (2021) discuss academic integrity in STEM education and the impact brought on by the move to online education delivery during Covid-19. Specifically, the authors refer to a call for action which places the focus on empathetic teaching, learning and policy approaches that focus on student learning and support commenting further:

…Academic integrity is not about avoiding misconduct or adhering to a set of regulations but rather about practicing ethical decisions daily in learning institutions to habituate the process (para. 14).

Herein lies the central rationale of (a) the development of the approach to academic integrity by Rabdan Academy and (b) the contribution an evaluation of the holistic approach as described, offers to the issues raised in the preceding literature discussion. The published literature associated with academic integrity, police education, law enforcement education and, by extension, criminology / justice students is sparse. Interestingly, the early work of Coston and Jenks (1998) explored academic dishonesty among criminal justice majors concluding the criminal justice major students were ‘aware of, have engaged in and plan to become involved in low, medium and high levels of academically dishonest behavior in the future’ (p.235). In 2015 Eriksson and McGee published a study of academic dishonesty amongst Australian criminal justice and policing university students at which time the authors note research had been conducted in the United States with criminology students. Eriksson and McGee (2015) compared the findings with research from other disciplines and countries finding relative similarity in predictors of academic misconduct. Further, the comments by Eriksson and McGee (2015) indicated no research existed specifically associated with academic dishonesty in police education programs. The work of Eriksson and McGee (2015) being the first in the professional domain of policing. The work of Stout (2011) in examining professional ethics and academic integrity in police education suggested the ramifications of academic misconduct by a police officer (and other professionals) were of such a significant consequence consideration should be given to teaching academic integrity in police education programs as professional ethics studies. This current study offers currency on the issue of academic integrity in the context of police education.

## Research methodology and data collection

To enable contextualisation of the following discussion of data collection tools, data analysis and results as applied to this research it is appropriate to present the rationale for utilising a case study approach to the research study. The seminal work of Yin (2003) suggests a researcher would use the case study method when they deliberately plan to cover contextual conditions on the understanding, they may be highly informative to the phenomenon under study. Merriam (2009) in discussing the selection of case studies, suggests this form of research methodology is particularly appropriate for exploring problems related to education practice. Similarly, the work of Simons (2009) offers a valuable rationale for the use of a case study in the context of this current study:

…[a] case study using qualitative methods in particular enables the experience and complexity of programs and policies to be studied in depth and interpreted in the precise socio-political contexts in which programs and policies are enacted; through closely describing, documenting and interpreting events as they unfold in the ‘real-life’ setting, it can determine the factors that were critical in the implementation of a program or policy and analyse patterns and links between them (p. 179).

Simons (2009) further suggests case studies are appropriate to enable dissemination of findings beyond the case to inform on decision-making, policy and practice.

The perspectives of Yin (2003), Merriam (2009) and Simons (2009) in reference to data collection methods have informed on the data collection for this research. Whilst Yin (2003) advocates for scientific methods reliant on collecting empirical data to test hypotheses and challenge hypotheses, this current research does not readily lend itself to the development of specific hypothesis or theory.

The seminal work of Robert Burns for understanding research (2000) proffers the case study is a *‘portmanteau* term’ (p.459) that in the main typically involves the observation of an individual unit. Appreciatively, the study of Rabdan Academy’s approach towards and the subsequent outcomes associated with managing academic integrity fall within this concept of a case study.

The data associated with this study has been drawn from previously collected data by the Institution for administrative purposes. Human Research Ethics approval to utilise the anonymised data was confirmed by the Rabdan Academy Research Ethics Committee. The data utilised in this study included end-of-term student satisfaction surveys (the surveys are a mandatory requirement within Rabdan Academy conducted by the Institutional Effectiveness Unit); Academy faculty survey designed to understand their experience with transition to online learning (secondary data for this paper); student course grade achievements; violations of academic integrity (number and format); analysis of the data collected over the terms prior to implementation of the emergency distance learning, during the period of distance learning and the application of a range of strategies to reduce academic integrity violations.

The strategies are discussed in detail in the case description and refer to workshops conducted for faculty and students, authenticity check interviews and Respondus Lockdown mechanisms. The data is secondary in nature and is collective, whereby no faculty member, individual student or class specific data is captured, rather it is whole of Academy per term data. Comparison of changes in the respective data is utilised to understand the impact of the academic integrity mitigation strategies. The following case description offers insight into the environment and circumstances under which developing an operational understanding and culture of academic integrity was applied at Rabdan Academy.

### Case description

The onset of the Covid-19 pandemic in the UAE led to urgency in managing the design and delivery of education through the online (internet) environment – a previously generally unfamiliar pedagogy in the UAE. Whilst more than 58% of Rabdan Academy faculty had working experience with online course delivery, the transition preparation was required to be achieved in a two-week period and involved:

- 14 dedicated workshops for faculty professional development in designing teaching and assessment materials for the online platforms were presented;
- Development and dissemination of an online assessment manual for faculty;
- Development and dissemination of detailed proctoring guidelines for faculty to support them with the reporting of incidents taking place during online examinations.
- Development and dissemination of detailed, bilingual proctoring guidelines for students to support them with using online assessment tools and raise their awareness about possible offences.

The student cohort of 631 received approximately 30 learning opportunities focused on supporting students to utilize the range of software programs (Specialized software in combination with the Moodle Learning Management System, Webex, MS Teams, Respondus Lockdown, Turnitin, and Screen-o-Matic). These software programs were either in place within the institution pre the pandemic (Covid-19) or readily available and commonly being adopted by academic institutions within the UAE. and

- 30+ group/individual sessions to prepare the students for the 100% online learning and assessment environment; and
- A set of 30 instructional videos were developed to support the workshops and build a resource collection.

Establishing confidence for faculty and students in the online learning environment was paramount and the number of workshops, instructional videos and documented guidelines were critical to the preparation approach (Davies and Al Sharefeen 2021).

In the assessment strategies applied during the Spring 2020 – Fall 2020 period, two key approaches were employed i.e., Respondus Lockdown software for online examinations and Turnitin text-matching software for assessments requiring written papers and presentations, e.g., case studies and short research papers. The selection of these two tools was premised on (a) commonality with other United Arab Emirates education institutions, (b) Turnitin was in place at the Academy, however, prior to Summer 2020 it was not a mandatory requirement for assessments to be submitted through Turnitin. Post Spring 2020, all written assessments (except for handwritten examination papers and where applicable laboratory or similar physical assessment activities) were mandated to be submitted through the Turnitin text-matching software.

Due to several academic misconduct incidents during Spring 2020 and Summer 2020, three actions were taken at the start of Fall 2020:

- Introduction of a detailed academic incident procedure that clearly defines face-to-face and online academic misconduct incidents, categorizes them into major and minor, and provides examples of incidents and disciplinary action to be taken for each incident based on its seriousness (major vs minor), category, and frequency (first, second, etc.,).
- Moving all written examinations from online to Rabdan Academy’s campus starting Fall 2020.
- Introduction of an authenticity interview whereby faculty are required to conduct a 5-minute interview with each student who submits an assignment through Turnitin.
- Requiring faculty members to increase the percentage of open book assessments to promote critical thinking and limit opportunities for academic misconduct.

A brief explanation of the number of academic misconduct incidents at Rabdan Academy offers contextualising and insight into the subsequent focus on academic integrity undertaken by the Academy. The deployment of online assessment tools introduced during the transition to online learning (in response to Covid-19 health and safety requirements) resulted in an increase in the number of academic misconduct incidents, particularly notable in the Spring and Summer terms of 2020 at the Academy. The number of incidents were 486 (18% per registration record) in Spring 2020; 282 (26% per registration record) in Summer 2020; 268 (10% per registration record) in Fall 2020; and 49 (2% per registration record) in Spring 2021 as reported by faculty members. Prior to the deployment of distance education (Spring 2020) RA recorded only 55 incidents between Fall 2017 and Fall 2019. During that period, using Turnitin for all written assessments was an expectation rather than a requirement and the Academy did not have clear assessment guidelines or a student discipline procedure. Worth noting here is that only 67 incidents in Spring 2020 were for plagiarism and suspicious behaviour while the remainder were for dress code violation, tardiness, and technical issues. These latter types of incidents were not normally recorded prior to Spring 2020. In Summer 2020, 193 were recorded for academic misconduct, the remainder being for dress code violation, tardiness, and technical issues. A possible contributing factor for the sudden increase is the fact that conducting a correct environment check using Respondus Lockdown browser for online (internet based) assessments was not recorded as an incident in Spring 2020 however, commencing in Summer 2020 it was recorded as an academic integrity incident. The number of incidents associated with the Respondus Lockdown browser environment check in Summer 2020 stood at 91. A reduction in the number of incidents in Fall 2020 coincided with the introduction of the new academic discipline procedure and moving examinations from online to Rabdan Academy campus. Birks et al. 2020, suggests an increase is expected in academic misconduct with increased motivation for students to pay others to do their assessed written work and the new opportunities provided by the switch to online assessment. Similarly, the work of Amzalag et al. 2021 identified that students are more likely to engage in integrity misdemeanours during online assessments.

The deployment of Respondus Browser Lockdown and Monitor, an online proctoring solution, commencing in Spring 2020 for the Academy, proved to be challenging for both students, faculty, and administrative staff. Such challenges including the time taken to review camera recordings of individual students taking the exams (the system automatically highlights if there is a break in the recording during the exam – flagging a potential violation by the student); internet connectivity breakdown; incomplete setup procedures by the student (potential error vs purposeful misconduct).

Several strategies were employed to mitigate the challenges associated with the use of Respondus Lockdown Browser and Monitor. First, a dedicated page was created for faculty members on Moodle including videos and manuals on how to deploy the tool on course shells and how to design online assessments. Second, workshops were held for both faculty and students to understand the process. Third, a dedicated faculty member was assigned to check each online examination to ensure compliance with the guidelines. Fourth, each proctored group (size between 15 and 25) was assigned to one faculty member who conducted a 10-minute briefing via MS Teams to students about the test to be taken and reminding of the use of the online proctoring system. Students received invitations to those sessions and were provided with the opportunity to ask any questions they had about the examination procedures. During the conduct of an examination, phone numbers were provided to students to call the dedicated help desk if they were experiencing internet connectivity issues. The help desk logged the student ID and time of the call to enable alignment with the checking of the footage of the student taken by the Respondus System during the examination to exclude it from any potential misconduct incident. The fifth and final step included provision of five IT support staff members made available during each examination timeslot (a ratio of 30 students per IT support staff member) to assist with any technical issues that may arise during examinations. Appreciatively, this process was labour intensive and resulted in a significant increase on the workload of faculty and administrative staff.

To avoid academic misconduct incidents, four actions were taken at the Academy in the commencement of Fall 2020:

- Introduction of a detailed academic incident procedure that clearly defines face-to-face and online academic misconduct incidents, categorizes them into major and minor, and provides examples of incidents, and disciplinary action to be taken for each incident based on its seriousness (major vs minor), category, and frequency (first, second, etc.).
- Transfer of all written examinations (i.e., mid-term and final exams) from online to Rabdan Academy’s campus commencing in the Fall 2020 term.
- Introduction of an authenticity interview whereby faculty are required to conduct a 5-minute interview with each student who submits an assignment through Turnitin.
- Requirement for faculty members to increase the percentage of open book examination formats.
- Introduction of detailed Assessment Guidelines that articulate clear expectations for online (internet based) and face-to-face assessments.

To offer a holistic and supportive approach to students and faculty regarding academic integrity, the Academy conducted twice weekly presentations for all students for the first 4 weeks of the Fall 2020 term. Workshops familiarizing and reinforcing the whole of Academy commitment to high standards of academic integrity were conducted at the commencement of the Fall 2020 term for faculty. In addition, copies of all presentations and guidance for academic referencing were uploaded to the online Moodle sites for all courses, undergraduate and graduate conducted in the Fall of 2020. Faculty undertook to support the students through referral and explanation of referencing at the time of releasing written assessments requiring submission through Turnitin portals. All students at the Academy study a course of English Language 2; within this course there are topics focused on academic writing and referencing protocols using the Academy-preferred American Psychological Association referencing standards.

The new student discipline and academic integrity procedure enforced during Fall 2020 defined academic integrity categories, clear courses of action for violation of academic integrity and the respective process depending on the level and frequency of the incident by a student. The procedure categorized incidents into major and minor, outlined a new process for investigation, and provided examples of offences and associated consequences based on seriousness and frequency. Additionally, the procedure provided an authority matrix for decision making to support the institution to take timely decisions.

## Results

The significant impact of combining the multiple initiatives to reduce academic misconduct became clear in the Spring 2021 term where the total number of incidents reduced to 2% of the Academy total student number in comparison to previous terms (See data in previous section).

It can be argued that students could have benefited from the increased awareness of the new procedures and their implications and the sustained emphasis on teaching and supporting appropriate referencing of written work. Following the dismissal of the largest number of students for academic misconduct in a given semester (Fall 2020) in the history of the Academy, the message became clear to students that the institution is committed to upholding high academic standards.

### Turnitin text-matching software implementation

A further factor that may have influenced a reduction in the frequency of academic integrity violation incidents in Spring 2021 was (a) enabling students to view the similarity percentage at the time of submission into Turnitin and permitting students to resubmit written assignments after the submission due date when similarity is high. The opportunity to resubmit was accompanied by the criteria/condition that a late submission policy is implemented. According to the late submission policy, late submissions are allowed within a maximum of 5 working days of the deadline of the assessment activity. The student grade is reduced by 10% for each late working day during the aforementioned 5-day period. If the assignment is submitted beyond the 5-day period, the student receives a grade of 0%.

### Authenticity interviews

The data suggests the authenticity interview process may have served as a deterrent to students for hiring ghost writers to author their essays and assignments as nil such incidents were reported in the Spring 2021 term. Whilst it is not feasible to specifically identify the precise influence of the introduction of Turnitin coupled with the authenticity interviews, the student assessment results at the Academy in Spring 2021 demonstrated an improvement in students’ understanding of the assignments they had submitted – indicating potentially more student work vs ghost writers employed in the writing of the assessments.

The downside of authenticity interviews is that they result in an increase in the workload of faculty members as they are required to meet with students who had submitted assessed written work individually and ask them a set of questions (see Table 1). This finding as reported by faculty members in response to a faculty survey conducted by the Academy to understand the challenges with implementation of online (internet) computer-based assessment tools. To counterbalance the impact, an amendment was incorporated in the authenticity interview procedure, commencing in Fall 2021 whereby faculty members select at random a sample of students (20%) to undergo the authenticity interview per written assessment.

**Table 1:** Authenticity interview questions

Student Identity (ID)	The student demonstrates understanding of the topic of the written work	The student provided a gist demonstrating understanding of the key ideas of the written work	The student is able to answer 3–5 questions about details included in the written work	The student is able to explain 3–5 key terms appearing in the written work.	The student demonstrates understanding of key feedback provided by the faculty member

**Fig. 1:**
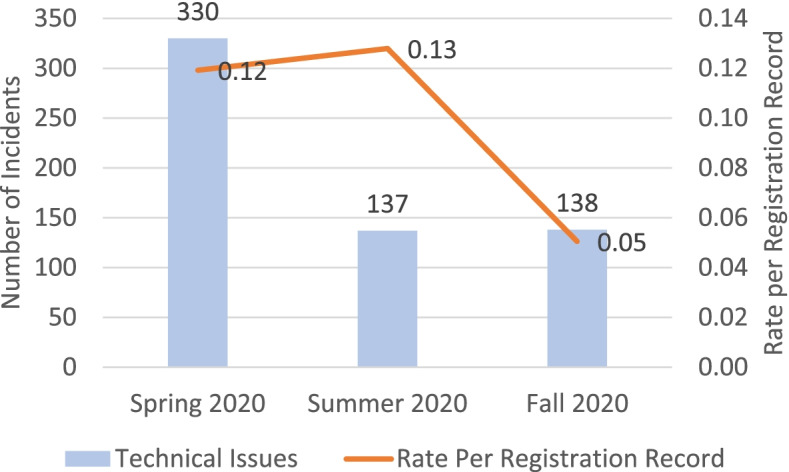
Technical issues with respondus lockdown per Academy student load

The implementation of the Respondus Lockdown online proctoring software produced a reporting of increased academic misconduct frequency. Approximately 30% of flagged potential violation incidents were associated with poor internet connectivity, which in many instances stopped the tool from recording the examination. As a result, up to 5% and 20% of examinations had to be re-administered (replacement examination for students) in Spring 2020 and Summer 2020, respectively, as the tool was not recording the examination accurately due to internet connectivity issues. At the same time, administering examinations online facilitated new opportunities for students to engage in unethical academic behaviours resonating with the findings in the work of Amzalag et al. 2021. For example, incidents of using a mobile phone, other persons being present and communicating with the examinee during the conduct of the assessment. Additionally, many students encountered technical issues during the deployment of Respondus Lockdown Browser and Monitor. Figure 1 shows that 330, 137, and 138 students reported having technical issues during the administration of exams in Spring 2020, Summer 2020 and Fall 2020, respectively. While some of these issues were resolved during the examination, others resulted in the re-administration (students required to undertake a replacement examination) of assessments. The tool significantly increased faculty workload as the system employed by the Academy did not enable live proctoring and faculty had to review a large volume of recorded content for individual students sitting examinations.

**Fig. 2:**
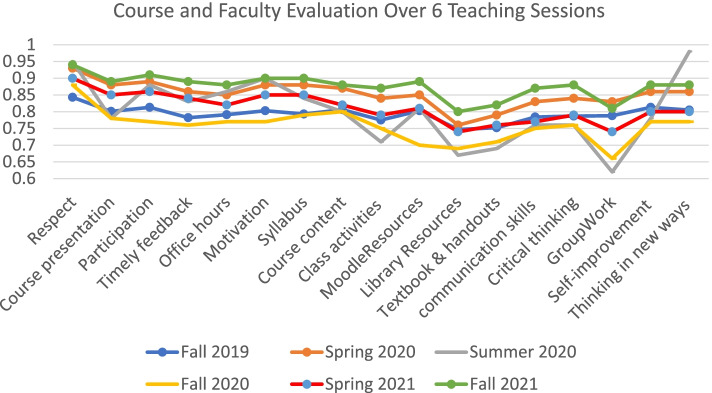
Course and Faculty Evaluations over 6 teaching sessions

As indicated in Fig. 2, the experience with the Respondus Lockdown coincided with a reduction in student overall satisfaction with the teaching during semesters in which teaching was delivered fully online (Summer 2020) as opposed to 10% of classes delivered on campus during Fall 2020 and 30% in Spring 2021. Spring 2020 should be treated as a unique semester in the context that students were not removed from courses for poor attendance, dismissed from the academic institution, or had their academic standing ranking impacted. These conditions were mandated by the Ministry of Education mandate (Resolution 237 of 2020). These conditions contribute to informing on the exceptionally high satisfaction rate of students in Spring 2020 compared to other teaching sessions. The data presented in Fig. 2 indicates students were least satisfied in Fall 2020. This level of dissatisfaction coincides with the introduction of the various controls in Fall 2020 to mitigate the risk of academic misconduct, moving exams to campus, and the abrogation of UAE Resolution 237 which, as indicated earlier, allowed students to continue despite having high absence rate and low performance (Spring 2020). The decreased satisfaction of students also manifested itself in the high exam absence rates and high-grade appeal requests during Fall 2020.

### On campus examinations

**Fig. 3:**
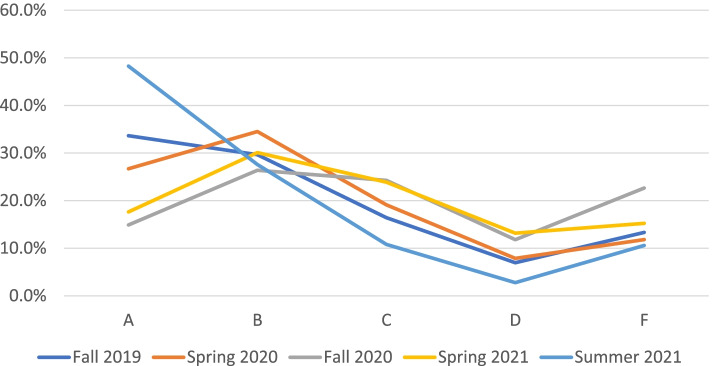
Grade distribution over 5 teaching sessions

The delay in identifying a tangible reduction in academic integrity violation incidents in Fall 2020 may be attributed to three key circumstances. First, planning for on campus examinations took more time than expected in light of Covid-19 health and welfare restrictions, resulting in conducting a majority of assessments in the second half of the semester. This delay, coupled with the introduction of the new discipline procedure and assessment guidelines, may have negatively impacted student results in Fall 2020 (see Fig. 3) and student satisfaction (Fig. 2). It is not unreasonable to suggest the circumstances i.e., compressed assessment periods, increased the pressure on students during a term which realised a higher rate of academic integrity violations. There are implications here that students have a higher tendency to commit academic offences during a condensed period where the frequency of assessments is higher than during a normal teaching session. The suggestion that the intense teaching periods and assessment/examination periods influence higher rates of academic integrity violations is further supported by the outcomes of the frequency of assessments is higher than that in regular teaching session. In Summer 2020 the number of incidents that can be clearly labelled as academic misconduct (suspicious behaviour, missing from camera frame, and plagiarism) were 66 (or 7% of registration records) compared with 60 (or 2% of registration records) in Spring 2020.

In Spring 2021 there was a noticeable reduction in the frequency of academic integrity incidents (18%, 26%, 10%, and 2% per registration record in Spring 2020, Summer 2020, Fall 2020 and Spring 2021 respectively). One of the reasons responsible for the reduction may be attributed to the increase in the duration of final examination periods ensuring that students do not have more than one examination per day (in previous teaching sessions students had occasions of 2 exams per day). This finding is supported by a number of studies on reasons for academic misconduct (see Amzalag et al. 2021; Birks et al. 2020; Amigud and Lancaster 2019; Bretag et al. 2019a, 2019b; Krou et al. 2021) where a large overload of studies is cited as the reason for committing academic misconduct. Commencing in Spring 2021 faculty were required not to administer online (internet-based) quizzes during the term. On exceptional cases online quizzes were approved provided that faculty integrated open book questions. Additionally, to address the increase in workload associated with distance learning during condensed periods, the Institution adopted a policy commencing in Summer 2021 of preventing students from enrolling in more than one course during condensed teaching sessions, unless the student is a graduating student.

## Discussion

As suggested in the work of Reedy et al. 2021a, 2021b; Khan et al. 2020, there is value in strategies for embedding institutional academic integrity through building a holistic faculty and student in unison approach. It is this approach taken by Rabdan Academy as discussed in this paper that has enabled the engagement of a number of strategies including student workshops, faculty workshops, reference materials, authenticity interviews and Turnitin and Respondus Lockdown software to build a whole of Academy approach to this important academic institutional responsibility. Whilst the concept of whole of institution approach is not new, (Reedy et al. 2021a, 2021b; Khan et al. 2020) the on-set of Covid-19 and the urgency to transition to remote delivery of learning through, in the main, internet-based platforms created opportunity to develop processes and protocols to mitigate potential avenues for student transgression of academic integrity. Rabdan Academy’s approach has sought to combine findings from the literature, for example closing a gap identified by Sefick et al., 2020 in providing student and faculty training on what constitutes academic misconduct and establishing policies and procedures that identify the action/s considered a transgression and the respective penalties. An important concept in the Rabdan Academy approach is the scaffolding of academic integrity transgressions into minor and major which, in the reality of application, allows students to learn from their mistakes. For example, a grade of 0 in an assessment vs a grade of 0 in the course. The transparency of the consequences of academic integrity was called for in the work of Sefcik et al. 2019 to ensure processes and procedures extended beyond referencing and plagiarism curricular. Here the central tenet is providing the learning opportunity whilst also demonstrating the level of seriousness with which Rabdan Academy views academic integrity. The strategies taken by Rabdan Academy for the issue of academic integrity has embraced the holistic (faculty and students) approach (Liang and Maddison 2021; Reedy et al. 2021a, 2021b; Thacker et al., 2020; Chankova 2020), supporting the student to be at the centre of the learning and activity (Freeman et al., 2014); and designing in authenticity interviews in the endeavour to mitigate cheating in written out-of-class assessments (Bretag et al. 2019a, 2019b) The overarching approach which combines the initiatives to address each of the avenues for academic integrity transgressions has resulted in early indications of reducing the instances of academic integrity transgressions.

## Conclusion

The development and application of initiatives targeting the mitigation of academic misconduct in educational institutions is an ongoing task. The factors which motivate students to conduct academic misconduct continue to evolve. Where institutions are able to readily identify the influencing factors, intervention strategies, policies and procedures are able to be developed and applied. It is the unknown influencing factors which continue to turn student performance into a harmful statistic both for the student and the organisation.

As indicated in the Rabdan Academy’s experience of iterative interventions and developments in the pursuit to reduce academic misconduct incidents, mitigation is a holistic and continuously evolving process. Of note, this approach is realizing tangible results for Rabdan Academy students with a progressive reduction in academic misconduct incidents. Commitment to continuous improvement of strategies informed by current experience and contemporary literature in this domain to embrace the ‘lessons learnt’ from the global education community offers a pathway to support students, faculty, and institutions in achieving high standards of academic integrity. The Rabdan Academy strategies developed and applied as a student and faculty combined approach included:

- Comprehensive learning and teaching program for faculty and students dedicated to academic misconduct;
- Comprehensive learning and teaching dedicated to student understanding academic referencing and text-matching protocols with continuous student support throughout the teaching terms;
- Comprehensive and widely publicized academic misconduct policies and procedures;
- Authenticity Interviews for out-of-class written assessments.

This model is replicable and the early positive trending outcomes offer insight in contributing to the global academic community endeavours to address academic integrity.

## Data Availability

The datasets during and/or analysed during the current study available from the corresponding author on reasonable request.
